# Prospective Analysis on Survival Outcomes of Nonsmall Cell Lung Cancer Stages over Ⅲb Treated with *HangAm-Dan*

**DOI:** 10.3779/j.issn.1009-3419.2010.11.03

**Published:** 2010-11-20

**Authors:** Tae-Young JEONG, Bong-Ky PARK, Yeon-Weol LEE, Chong-Kwan CHO, Hwa-Seung YOO

**Affiliations:** East-West Cancer Center, College of Oriental Medicine, Daejeon University, Daejeon, Korea

**Keywords:** Lung neoplasms, *HangAm-Dan*, Oriental medicine, Overall survival rate, Cancer, Herb

## Abstract

**Background and objective:**

Non-small cell lung cancer (NSCLC) stages over Ⅲb still remain as an intractable disease. Survival rate of NSCLC stages over Ⅲb could be increased through chemotherapy and radiation, but results are not satisfactory. Oriental medicine herbal formula, *HangAm-Dan* (HAD) has been developed for anti-tumor purpose and several previous studies have already reported its effects. The aim of this study is to assess HAD's efficacy on prolonging the survival rate of NSCLC stages over Ⅲb.

**Methods:**

We have administered 3 000 mg of HAD daily to patients. The study included 74 first visit patients of East-West Cancer Center (EWCC) from November 2007 to April 2008, diagnosed with inoperable NSCLC stages over Ⅲb. Among them, 30 patients were in HAD group and 44 patients were in combined group with conventional therapy and HAD. We have observed and analyzed their overall survival.

**Results:**

Of total 74 patients, overall 1 year, 2 year survival rates and the median survival time were 62.1%, 34.9% and 17.0 months (95%CI: 12.9-21.1). NSCLC stage Ⅲb patients showed higher survival rates than NSCLC stage Ⅳ patients (*P*=0.408). The 1 year, 2 year survival rates and the median survival time of the combined group were 70.5%, 37.9% and 20.0 months (95%CI: 16.4-24.6). In HAD group, the 1 year, 2 year survival rates and the median survival time were 50.0%, 25.7% and 12.0 months (95%CI: 6.6-17.4). The combined therapy group showed higher survival rates than the HAD group (*P*=0.034). Each groups treated with HAD for more than 4 weeks showed higher survival rates than those treated for less than 4 weeks, but there was no significant difference (*P*=0.278). In hazard ratio, the combined therapy group showed lower mortality rate than the HAD group with statistical significance (*P*=0.040).

**Conclusion:**

HAD could prolong the survival rate of inoperable NSCLC stages over Ⅲb. HAD is more effective when combined with conventional therapy. In the future, more controlled clinical trials with larger sample in multi-centers are needed to reevaluate the efficacy and safety of HAD.

## Introduction

According to 2008 cancer statistics of International Agency for Research on Cancer of World Health Organization (WHO), lung cancer is the major cancer with highest incidence (12.7%) and mortality (18.2%) rate worldwide^[1]^. According to 2007 statistics from Korean National Cancer Center, lung cancer was ranked 4^th^ (11.0%) highest in cancer incidence of Korea. Especially in male population, lung cancer was ranked 2^nd^ (15.1%) followed by stomach cancer (20.3%). In 2006 cancer mortality, lung cancer ranked 1^st^ place (14.8%) in Korea^[2]^. Moreover, in cancer statistics of United States 2009, lung cancer ranked 1^st^ place for both incidence (14.8%) and mortality (28.3%) rate^[3]^. Cancer statistics of Europe 2008 has also conveyed lung cancer to be 3^rd^ highest in incidence (12.2%) and 1^st^ in mortality (19.9%)^[4]^. Markedly, mortality rate is relatively higher than that of incidence which makes lung cancer to be one of the most intractable cancers.

Lung cancer is mainly classified into 2 groups, non-small cell lung cancer (NSCLC) and small cell lung cancer (SCLC)^[5]^. Reason behind this classification of two groups is due to the differences in treatment and prognosis. Approximately 80% of total lung cancer patients make up NSCLC patients^[6]^. NSCLC consists of 3 cell types of lung cancer; adenocarcinoma, squamous cell carcinoma, and large cell carcinoma^[5, 6]^. Treatment of NSCLC is decided by stages of tumor. Generally, treatment for stages Ⅰ and Ⅱ are surgical resection and adjuvant chemotherapy. In case of stage Ⅲa, general treatment is neoadjuvant chemotherapy followed by surgical resection or chemoradiation. However, stage Ⅲb or Ⅳ NSCLC patients are most likely to be inoperable and are treated by chemotherapy or chemoradiation^[7]^.

*HangAm-Dan* (HAD) was developed by East-West Cancer Center (EWCC) in 1996 for anti-tumor purpose. Based on the experimental research of its efficacy and safety, the prescription had been modified. HAD consists of 9 anti-tumor oriental medicine herbs. Anti-tumor effects and safety of HAD have been proven by both *in vitro* and *in vivo* studies^[8-11]^. Moreover, reports on Wheel Balance Therapy (WBT), a traditional Korean medical therapy using HAD had shown positive case studies and retrospective studies on lung cancer as well^[12-16]^.

But as case studies and retrospective studies have many limitations, prospective study was developed to confirm antitumor effects of HAD. Based on the results of previous studies, this prospective study is to investigate anti-tumor effects of HAD in inoperable NSCLC patients over stages Ⅲb.

## Patients and Methods

### Eligibility

From November 2007 to April 2008, 121 lung cancer patients who visited EWCC for the first time were enrolled in this study.

Eligibility criteria were as follows: (1) Diagnosis of histologically or cytologically proven lung cancer; (2) Nonsmall cell lung cancer (inoperable stages of Ⅲb or Ⅳ); (3) ECOG performance^[17]^ 0-2; (4) At least one time of patients followed up; (5) More than one day of HAD administration; (6) Life expectancy of more than 1 month.

121 lung cancer patients visited EWCC from November 2007 to April 2008 for the first time. By eligibility criteria, 47 patients were excluded and 74 patients were selected in this study (Fig 1). All patients provided written informed consent. Institutional review boards have approved the trial protocol before the patient enrollment.

**1:**
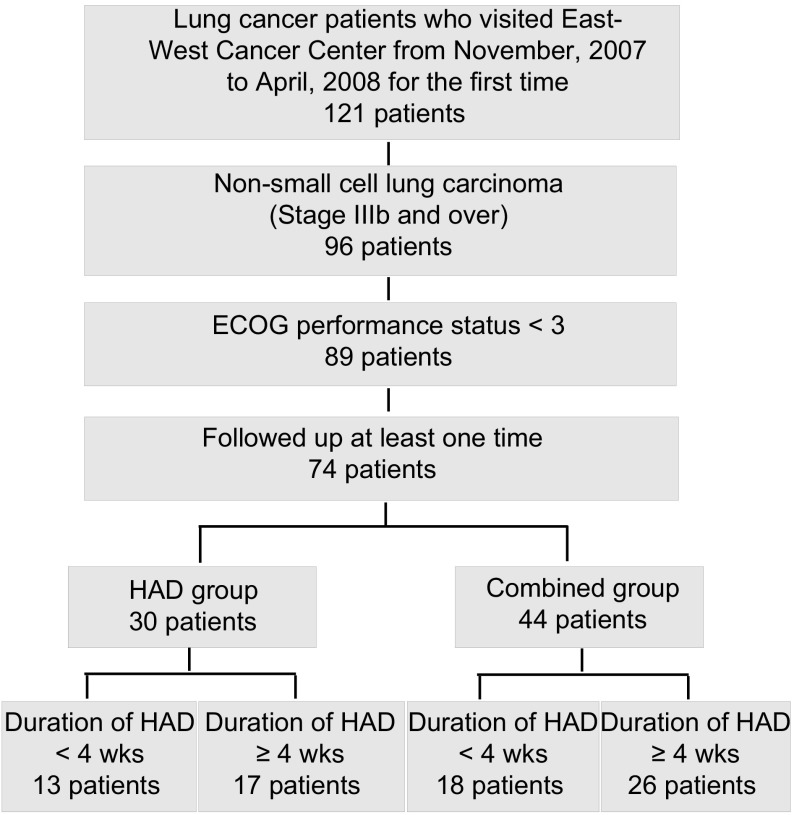
The flowchart for selection and classification of patients. Total of 74 patients were selected by eligibility criteria and classified into 4 groups.

### Treatment groups

We classified patients into two groups, HAD group and combined group treated with HAD and concurrent conventional therapy. Patients of both groups were given 3 000 mg of HAD daily. (1) HAD group. Patients of this group were treated with HAD without conventional cancer therapy during the enrolled period. (2) Combined group. Patients of this group were given HAD combined with conventional cancer therapy. Combined group received concurrent or sequential conventional cancer treatment more than once during HAD administrations.

Among 74 patients, 30 patients enrolled in HAD group and 44 patients enrolled in combined group. Each group was then divided by the duration of HAD treatment (< 4 weeks or ≥4 weeks) (Fig 1).

### Prescription of HAD

HAD is the name of an anti-cancer herbal prescription. HAD consists of 9 herbs (Tab 1). HAD comes in capsules of 500 mg each. HAD is usually taken 3 times a day, 1 000 mg at a time, after meals (total 3 000 mg/d).

**1:** Prescription of *HangAm-Dan*

Scientific name	Amount (mg)
Coisis semen	259.0
Panax notoginseng radix	86.0
Hippocampus kelloggii	26.0
Cordyceps militaris	26.0
Santsigu tuber	26.0
Ginseng radix	26.0
Bovis calculus	17.0
Margarita	17.0
Moschus	17.0
Total amount (1 capsule)	500.0

### Assessment items and statistical analysis

(1) Basic characteristics. Investigated items are as follows: gender, stage, age, histopathology, metastasis, undergone conventional treatment, types of treatment (HAD group, combined group), treatment duration (< 4 weeks or ≥4 weeks). And adverse effects [symptoms, hematologic toxicity, hepatotoxicity and nephrotoxicity based on Common Terminology Criteria for Adverse Events (CTCAE) version 3.0] ^[18]^ during HAD treatment were also investigated. (2) Overall survival (OS) and hazard ratio. OS was calculated from the day of diagnosis to death. We estimated overall survival and median survival (months, 95%CI) according to stage, treatment types and duration by *Kaplan-Meier* method. And we also estimated hazard ratio based on the results of treatment type and duration by *Cox* regression. *P* < 0.05 was considered statistically significant.

## Results

### Patients characteristics

Characteristics of patients are shown in Tab 2. Relatively larger number of male patients participated than female patients (53 *vs* 21). Mean age was 63.3±11.5 years. Eight patients were stage Ⅲb (10.8%) and 66 patients were stage Ⅳ (89.2%). The median duration from diagnosis to HAD treatment was 9.0 months. The median duration of HAD treatment was 1.0 month.

**2:** General characteristics of 74 patients

Characteristics		Number (%) Median (95%CI)
Gender	Female : Male	21 (28.4) : 53 (71.6)
Age	Mean±SD	63.3±11.5
Stage	Ⅲb : Ⅳ	8 (10.8) : 66 (89.2)
Duration from diagnosis to HAD treatment		9.0 months (5.0-13.0)
Duration of HAD treatment	< 4wks	1.0 month (0.5-1.5) 31 (41.9)
	≥4wks	43 (58.1)
Combination with chemotherapy during HAD treatment	Yes	44 (59.5)
	No	30 (40.5)
HAD group	Age (Mean±SD)	67.7±11.2 4
	Gender (Female : Male)	4 (13.3) : 26 (86.7)
	Stage (Ⅲb : Ⅳ)	4 (13.3) : 26 (86.7)
Combined group	Age (Mean±SD)	60.2±10.2
	Gender (Female : Male)	17 (38.6) : 27 (61.4)
	Stage (Ⅲb : Ⅳ)	4 (9.1) : 40 (90.9)
CI: Confidence interval; SD: Standard deviation; HAD: *HangAm-Dan*; wks : weeks.		

### Survival

#### Overall survival of total patients

At the time of analysis, 55 patients (74.3%) were deceased and 19 patients (25.7%) survived. OS are shown in Fig 2.

**2:**
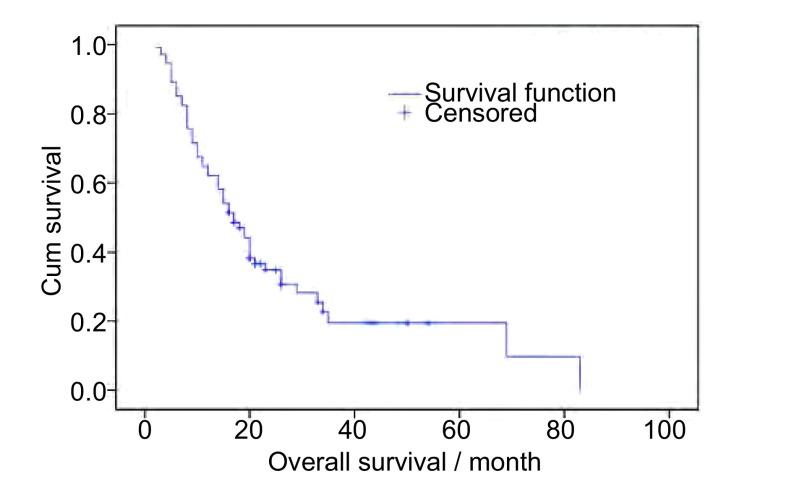
Overall survival of total patients. Median survival of all patients was 17.0 months. Overall survival rate was 62.1% for 1 year and 34.9% for 2 year respectively.

#### Overall survival by stage, treatment group and duration respectively

By stage, median survival of stage Ⅲb (*n*=8) and stage Ⅳ (*n*=66) were 20.0 months and 16.0 months respectively. OS of stage Ⅲb and stage Ⅳ were 75.0% and 60.6% for 1 year, 50.0% and 33.0% for 2 year respectively, but there was no statistical significance (*P*=0.408).

By treatment group, median survival of HAD group (*n*=30) and combined group (*n*=44) were 12.0 months and 20.0 months respectively. OS of HAD group and combined group were 50.0% and 70.5% for 1 year, 25.7% and 37.9% for 2 year respectively. It was statistically significant (*P*=0.034).

By treatment duration, median survival of under 4 weeks group (*n*=31) and over 4 weeks group (*n*=43) were 15.0 months and 19.0 months respectively. OS of under 4 weeks group and over 4 weeks group were 61.3% and 67.4% for 1 year, 27.5% and 39.9% for 2 year respectively, but there was no statistical significance (*P*=0.278) (Fig 3).

**3:**
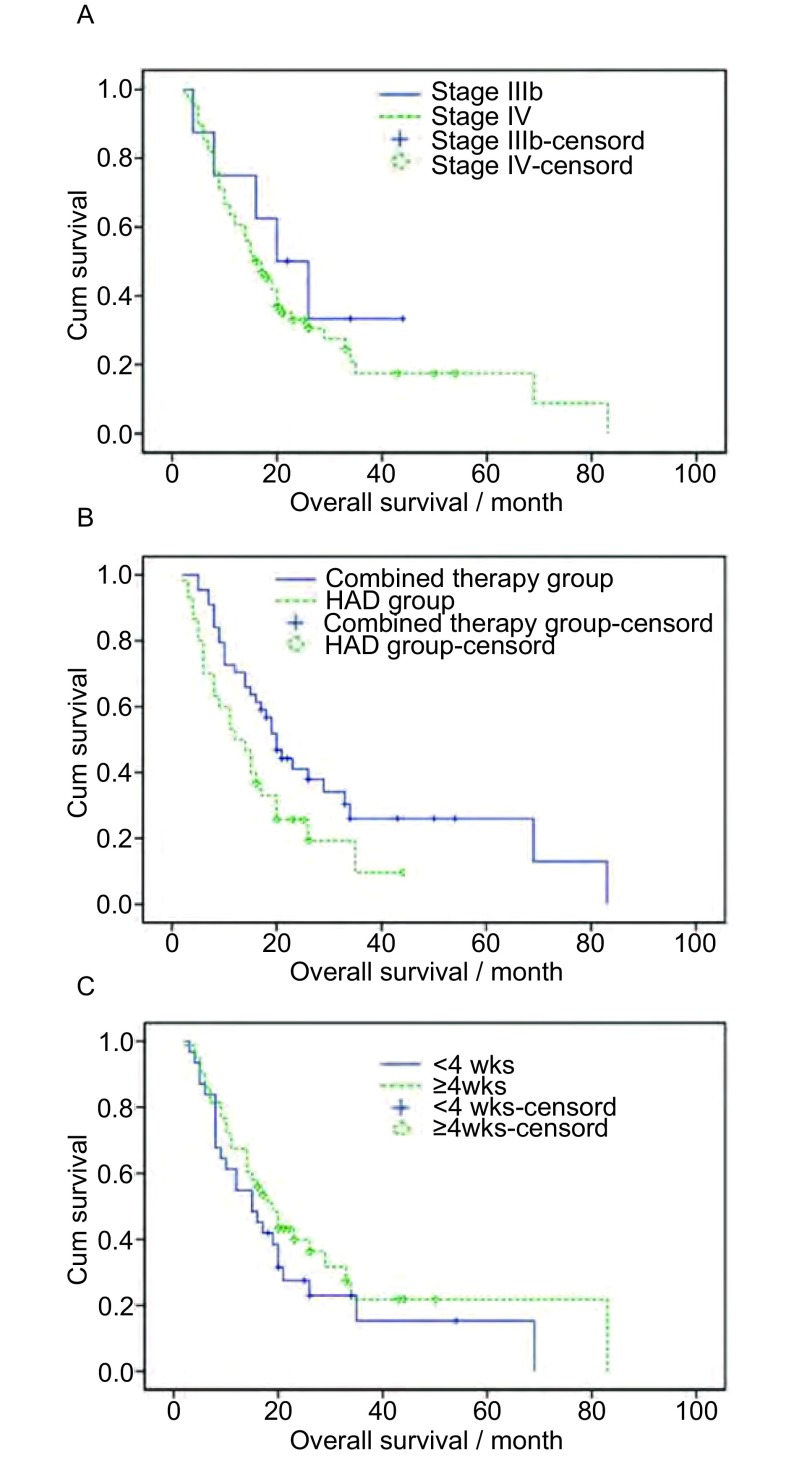
Overall survival by (A) stage, (B) treatment group and (C) duration. Overall survival (OS) of stage Ⅲb, combined group, over 4 weeks group were longer than stage Ⅳ, HAD group, under 4 weeks group respectively. OS by treatment group was statistically significant (*P* < 0.05), but other results (by stage, treatment duration) were not statistically significant.

#### Overall survival and hazard ratio by treatment group and duration

OS by treatment group and duration are shown in Tab 3. Combined therapy group with duration of HAD over 4 weeks showed the highest median survival, 1 year and 2 year survival rate. But it was not statistically significant (*P*=0.134) (Tab 3).

**3:** Overall survival by treatment group and duration

Treatment groups	Duration of HAD treatment	*n*	Censored	Median (95%CI)	1 year survival rate (%)	2 year survival rate (%)	*Log-rank P*
HAD group	< 4 wks	13	2	12.0 months (3.8-20.2)	46.2	23.1	0.134^*^
	≥4 wks	17	4	14.0 months (4.9-23.1)	52.9	28.2	
Combined therapy group	< 4 wks	18	4	16.0 months (8.2-23.8)	61.1	30.0	
	≥4 wks	26	9	23.0 months (10.6-35.4)	76.9	47.8	
^*^P < 0.05 was considered statistically significant.							

In hazard ratio, combined therapy group showed lower mortality with statistical significance. Over 4 weeks group showed lower mortality than under 4 weeks group without statistical significance (Tab 4).

**4:** Hazard ratio by treatment groups and duration

		Exp(B)	95%CI	*P*
Treatment groups	HAD group	1	0.328-0.974	0.040^*^
	Combined group	0.565		
Treatment duration	< 4 wks	1	0.438-1.279	0.289
	≥4 wks	0.748		
^*^P < 0.05 was considered statistically significant.				

#### Safety

No HAD related hematologic toxicity, hepatotoxicity and nephrotoxicity were observed. No non-hematologic HAD related adverse reactions were observed. No patients discontinued treatment due to any HAD related adverse events.

## Discussion

This study was designed to investigate anti-tumor effect of HAD in advanced NSCLC stages over Ⅲb. In inoperable NSCLC stages over Ⅲb, conventional therapy is chemotherapy. In this case, standard first line anticancer agents given were platinum based doublet chemotherapy. Paclitaxel, gemcitabine, vinorelbine, docetaxel, pemetrexed, *etc*. were combined with platinum chemotherapy agents. Docetaxel, pemetrexed, gefitinib, erlotinib, *etc*. were selected as over second line chemoagents^[19-21]^.

In advanced cancer clinical trials, commonly used criteria for anticancer drug are progression-free survival (PFS), response, failure-free survival (FFS), time to progression (TTP) and overall survival (OS). Among them, OS is the gold standard endpoint in advanced cancer clinical trials for anticancer drugs^[22-25]^. Moreover, HAD targets inhibition of angiogenesis^[9]^, and as mentioned in introduction, several case studies report HAD initiating tumor dormancy^[12-14]^. For these reasons, we have decided the OS to be most appropriate indicator for demonstrating HAD's anti-cancer effect than any other index.

We investigated previous clinical studies reported after 2006 on inoperable patients, stages over Ⅲb. In most studies, chemotherapies are classified into first line and over second line. In cases of first line chemo, chemoagents are mainly platinum based doublet therapy. And studies on gefitinib or docetaxel plus s-1 are also reported. In clinical studies of first line chemotherapy, median survivals are 8.0-18.6 months^[26-38]^. In cases of over second line chemotherapy, chemoagents are docetaxel, erlotinib, gefitinib, pemetrexed, *etc*. and median survivals are 5.9-13.4 months^[39-43]^. In this study, median survivals of HAD group and combined group were 12.0 months and 20.0 months, respectively. Median survival of patients who were treated over 4 weeks was 14.0 months for HAD group, and 23.0 months for combined group. Although results of this study can not be directly compared with results of other studies, OS of combined group was greater than that of the other studies.

In hazard ratio, difference of survival between HAD and combined group was statistically significant. So we can presume that HAD treatment with chemotherapy was more effective than HAD only treatment. The result was in agreement with that of the previous retrospective cohort study on WBT, which is a traditional Korean medical therapy using HAD^[15]^. The treatment duration results were not statistically significant.

In safety aspect, patients who were treated with HAD did not report any hematologic and non-hematolog adverse reactions. On the other hand, most chemoagents have toxicity, which in many cases lead patients to stop or delay their treatments^[44]^. In this study, no patients have discontinued treatment due to HAD related adverse events.

In this study, HAD treatment combined with conventional chemotherapy showed longer survival time than previous clinical studies with no significant adverse events. But this study may have several limitations to prove the effect of HAD, as follows: (1) Small numbers of patients; (2) Short HAD treatment period; (3) Each patients having different treatment history; (4) No consideration of the gap between initial diagnosis time and HAD starting time in calculation of survival; (5) No classification of first line and over second line chemotherapy.

In conclusion, HAD is worth investigating as an alternative natural anticancer agent for advanced NSCLC, but its effect has not been confirmed clearly yet. We also conclude that this study was a major step forward as a prospective study on HAD compared to previous case studies and retrospective studies. In the future, more controlled clinical trials with larger sample in multi-centers are needed to re-evaluate the efficacy and safety of HAD.
